# Ce^4+^-Substituted Ni–Al mixed oxide: fluoride adsorption performance and reusability

**DOI:** 10.1039/d3ra07690c

**Published:** 2024-01-03

**Authors:** Ararso Nagari Wagassa, Amit Bansiwal, Tofik Ahmed Shifa, Enyew Amare Zereffa

**Affiliations:** a CSIR-National Environmental Engineering Institute Nehru Marg Nagpur 440020 India; b Department of Applied Chemistry, Adama Science and Technology University, Adama P.O. Box 1888 Ethiopia enyew.amare@astu.edu.et; c Department of Molecular Science and Nanosystem, Ca’ Foscari University Venice Via Torino 155 30172 Venezia Mestre Italy

## Abstract

In this study, Ce^4+^-doped Ni–Al mixed oxides (NACO) were synthesized and comprehensively characterized for their potential application in fluoride adsorption. NACOs were examined using Transmission Electron Microscopy (TEM) and Scanning Electron Microscopy (SEM), revealing a sheet-like morphology with a nodular appearance. X-ray diffraction (XRD) analysis confirmed the formation of mixed oxides of cubic crystal structure, with characteristic planes (111), (200), and (220) at 2*θ* values of 37.63°, 43.61°, and 63.64°, respectively. Further investigations using X-ray Photoelectron Spectroscopy (XPS) identified the presence of elements such as Ni, Al, Ce, and O with oxidation states +2, +3, +4, and −2, respectively. The Brunauer–Emmett–Teller (BET) analysis indicated that NACO followed a type IV physisorption isotherm, suggesting favorable surface adsorption characteristics. The adsorption kinetics was studied, and the experimental data exhibited a good suit to both pseudo-first order and pseudo-second order, as indicated by high *R*^2^ values. Moreover, the Freundlich isotherm model demonstrated a good fit to the experimental data. The result also revealed that NACO has a maximum capacity for adsorption (*q*_max_) of 132 mg g^−1^. Thermodynamic studies showed that fluoride adsorption onto NACO was feasible and spontaneous. Additionally, NACO exhibited excellent regeneration capabilities, as evidenced by a remarkable 75.71% removal efficiency at the sixth regeneration stage, indicating sustained adsorption capacity even after multiple regeneration cycles. Overall, NACOs displayed promising characteristics for fluoride adsorption, making them potential candidates for efficient and sustainable water treatment technologies.

## Abstract 2

In this study, Ce^4+^-doped Ni–Al mixed oxides (NACO) were synthesized and comprehensively characterized for their potential application in fluoride adsorption.

## Graphical abstract

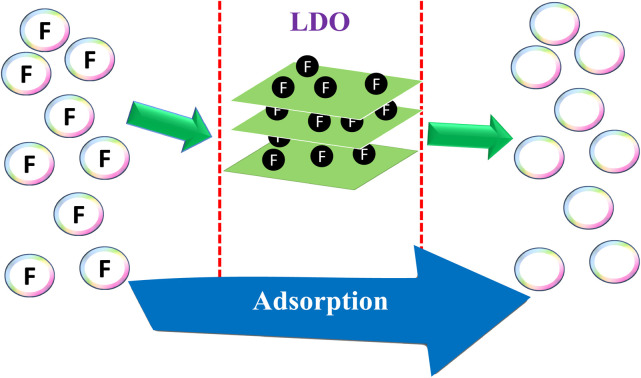

## 1. Introduction

Fluoride contamination is a significant global issue, particularly in developing countries, as it has detrimental effects on groundwater quality and poses serious health risks to humans, including dental and skeletal fluorosis.^1^ To address this problem, various methods such as adsorption, coagulation, precipitation and membrane filtration have been proposed for fluoride (F^−^) removal. Among these techniques, adsorption is widely recognized as one of the most effective and cost-efficient approaches for defluoridation.^2^ Therefore, the on-going search for a suitable adsorbent material to remove fluoride from water is of utmost importance. It is crucial to develop an efficient and affordable material to eliminate fluoride from water.

The search for an appropriate adsorbent material involves exploring natural and synthetic substances that have the ability to bind fluoride ions. Natural adsorbents like activated alumina, bone char, and zeolites have been extensively studied and utilized for fluoride removal, showing promising results in reducing fluoride concentrations in water. However, these materials may have limitations in terms of cost, availability, or regeneration capabilities.^3^

In recent years, developing mixed oxides, a class of materials with a combination of two or more metal oxides, have garnered significant attention due to their remarkable properties and diverse applications. These materials offer synergistic advantages over their individual oxide counterparts, exhibiting enhanced catalytic activity, improved stability, and tailored electronic and optical properties. They have demonstrated exceptional performance in a wide range of applications, particularly in water treatment and photo-/electrocatalysis. For instance, CuO_*x*_@Co_3_O_4_ core–shell nanowires exhibited remarkable sensitivity, selectivity, and response time in glucose oxidation, making them promising candidates for non-enzymatic glucose sensors.^4^ Similarly, MOF-on-MOF-derived hollow Co_3_O_4_/In_2_O_3_ nanostructure, showcased superior photocatalytic CO_2_ reduction efficiency, highlighting their potential in sustainable energy applications.^5^ Mg–Al layered double hydroxide (LDH) derived mixed oxide with enhanced fluoride removal capacity (222 mg g^−1^) was reported.^6^ LDHs, with their layered structure and exchangeable interlayer cations, serve as excellent precursors for the fabrication of mixed oxides. Calcination of LDHs leads to the decomposition of the hydroxide layers, resulting in the formation of mixed oxides with well-defined structures and compositions. This method offers several advantages, including ease of synthesis, high crystallinity, and control over the morphology and particle size of the mixed oxides.^3^

The remarkable performance of mixed oxides derived from LDHs stems from their unique structural and compositional features. The intimate contact between different metal oxides within the mixed oxide structure facilitates synergistic interactions, leading to enhanced electron transfer and catalytic activity. Additionally, the presence of multiple metal ions with different oxidation states and electronic properties can modulate the redox potential of the mixed oxide, further improving its catalytic performance. Moreover, calcined layered double hydroxides (CLDHs) can adsorb different anions by reconstructing their original LDH structure through the memory effect.^3^

In line with these advancements, the present study proposes Ce^4+^-doped Ni–Al mixed oxides as a novel fluoride adsorbent material. By introducing Ce^4+^ dopants into the Ni–Al mixed oxide structure, electron-deficient Ce^4+^ sites are created, activating the surface hydroxyl groups and resulting in an enhanced fluoride adsorption capacity.

To further elaborate on the significance of this study and considering actual applications in real world, investigating the influence of various factors on fluoride removal efficiency using Ce^4+^-doped Ni–Al mixed oxides is crucial. These factors include Ce^4+^ dopant concentrations, pH levels, initial fluoride concentration, adsorbent dosage, temperature, and contact time. By systematically studying these parameters, a comprehensive understanding of their impact on fluoride removal efficiency can be obtained, aiding in the optimization of the defluoridation process.

In summary, the study proposes Ce^4+^-doped Ni–Al mixed oxides as a novel adsorbent material for fluoride removal, leveraging the creation of electron-deficient Ce^4+^ sites and activation of surface oxides to enhance fluoride adsorption capacity. By investigating various factors and regenerability, the study contributes to the advancement of an efficient and cost-effective adsorbent for removing fluoride from aqueous solutions.

## 2. Experimental section

### 2.1 Synthesis of Ce-doped Ni–Al MOs

For the synthesis of Ce-doped Ni–Al mixed oxides, the following chemicals were used: nickel(ii) chloride [NiCl_2_·6H_2_O, 97%, LOBA CHEMIE PVT. LTD], aluminum chloride [AlCl_3_·6H_2_O, 98%, CDH(P) Ltd], and ammonium cerium(iv) nitrate [Ce(NH_4_)_2_(NO_3_)_6_, 0.1 M, INDETA Chemicals (India) Pvt. Ltd]. Merck provided sodium hydroxide (NaOH), potassium fluoride (KF) and hydrochloric acid (HCl). All of these compounds were analytical reagent grade and did not require further purification while being used. The solutions used in the experiment were made using type I deionized water (DI).

The Ce-doped Ni–Al mixed oxide adsorbents were obtained by calcination of the corresponding LDH at 450 °C using a microwave furnace for 2 hours. Initially, the precursor layered double hydroxide (LDH) containing different amounts of the dopant (at 5%, 10% and 20% of Ce to substitute Al) were synthesized following the method reported by Wagassa *et al.*^7^ The synthesis process of LDH was conducted under controlled pH conditions of 10 ± 0.5. Finally, the obtained MOs are coded as NACO-0, NACO-5, NACO-10 and NACO-20 indicating the amount of dopant.

### 2.2 Characterization

The Ce-doped Ni–Al MOs were thoroughly characterized using various techniques. Scanning Electron Microscopy (SEM, a TESCAN VEGA3 equipment) and High-Resolution Transmission Electron Microscopy (HRTEM, a JEOL JEM 2100 PLUS) were used to analyze the surface morphology. The elemental composition of the MOs was determined by Energy Dispersive X-ray Spectroscopy (EDS). The oxidation states of elements in the surface layer of MOs were measured with X-ray Photoelectron Spectroscopy (XPS) of an Oxford Instrument Omicron ESCA. X-ray powder diffraction (XRD) patterns were produced by scanning over the 2 range of 10 to 90° at a constant scanning rate with an X-ray diffractometer (Rigaku Miniflex-600) equipped with a copper Kα source. The VERTEX70 spectrometer (BRUKER, Germany) was used to record the spectra of attenuated total reflection Fourier transform infrared (ATR FT-IR) from 400 to 4000 cm^−1^.

### 2.3 Adsorption experiment

A batch adsorption technique was used to evaluate the efficiency of the synthesized MOs under different experimental conditions. Initially, the adsorption efficiencies of Ce-doped Ni–Al MOs were compared to identify the most effective MO for further study. Several factors such as interaction time, initial F^−^ concentration, adsorbent amount, agitation rate, pH of solution and temperature were explored. The contact time ranged from 15 to 120 min to examine the adsorption kinetics. Solution pH was varied from 3 to 11 to assess its impact on the adsorption process. Adsorbent doses (0.25 to 1.25 g L^−1^) were tested to determine the optimal amount for fluoride removal. The initial F^−^ concentration was varied from 10 to 60 mg L^−1^ to investigate the adsorption capability. Agitation rates from 50 to 250 rpm were used to study the effect of rate of mixing. Finally, the temperature was adjusted from 25 to 55 °C to evaluate its influence on the adsorption process.

After each adsorption process, the fluoride ion concentration was determined after filtering the samples through a 0.45 μm nylon syringe filter. A fluoride ion selective electrode was used to determine the filtered samples. Subsequently, the equilibrium adsorption capacity (*q*_e_) and present removal (*R*) were calculated using the equations below:^7^1
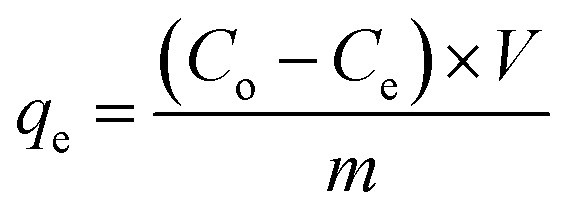
2
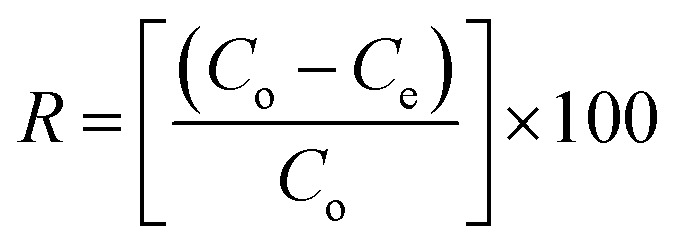
In these equations, *C*_o_ represents initial F^−^ concentration, *C*_e_ represents equilibrium F^−^ concentration, *V* denotes solution volume, and *m* indicates mass of the adsorbent.

## 3. Results and discussions

### 3.1 NACOs characterization

**Fig. 1:**
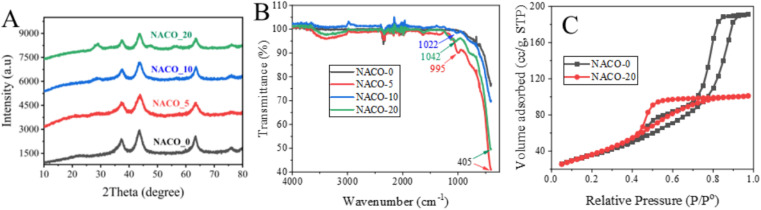
(A) XRD patterns, (B) ATR FT-IR spectrum and (C) BET characteristic of NACOs.

The as-prepared NACOs were analyzed using XRD. The obtained results are displayed in Fig. 1A. The XRD patterns revealed distinct diffraction peaks at specific angles of 37.63°, 43.61°, and 63.64°, which corresponded to the crystallographic planes (111), (200), and (220), respectively. These crystallographic planes were identified to correspond to NiO (JCPDS: 01-1239).^8,9^

The presence of well-defined diffraction peaks that corresponded to crystallographic planes (111), (200), and (220) suggested the formation of a well-defined crystal structure for the NACOs at lower concentration of the Ce dopant. However, as the amount of Ce increases distinct peaks typically associated with cerium oxide appeared at 29.0° (CPDS no. 34-0394).^10^ The XRD analysis provides valuable insights into the crystallography and phase impurity of the NACOs, as evidenced by the diffraction pattern when the amount of dopant increases.^8^

Average lattice constants of the synthesized NACO mixed oxide were determined from the planes (111), (200) and (220) which are the characteristics of the cubic system of NiO using eqn (3).^11^ The obtained results are depicted in Table 1.3
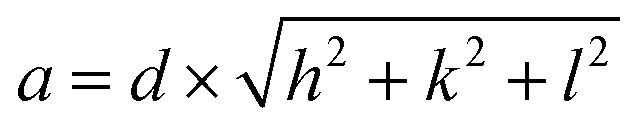
where a is distance between atoms in crystal, *d* is lattice spacing (inter-atomic spacing) and (*hkl*) is Miller indices. From the results, it can be observed that the lattice parameters of NACOs are ranged from 4.142 Å to 4.155 Å. These values are lower than that of pure NiO lattice parameter signifying the presence of Al^3+^ and Ce^4+^ ions in NiO nanoparticles.^12^ Thus, these XRD findings indicate that mixed metal oxides with a NaCl-type crystal structure were achieved through the thermal decomposition of the respective hydrotalcite-like compounds. As it is a simple cubic structure in which the metal cations and anions are arranged in alternating layers.^13^

**Table 1:** Crystallographic properties of NACOs

Materials	Lattice parameter (Å)	Average crystallite size (nm)
NACO-0	4.155	4.7
NACO-5	4.142	3.3
NACO-10	4.147	4.7
NACO-20	4.145	4.7

Average crystallite sizes of NACOs were calculated based on Debye–Scherrer equation from the three prominent XRD peaks of the materials. The determined results are presented in Table 1. As indicated in the Table 1 the crystallite sizes of all the materials are the same except NACO-5 with a slight change and this suggests that the introduction of Ce^4+^ ions did not significantly affected the crystallite size of NACOs.^14^4
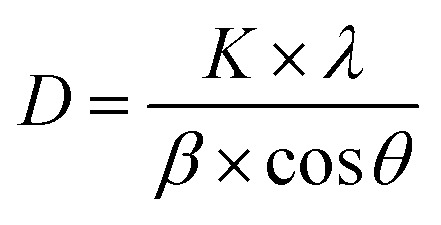
where *λ* is XRD wavelength (Cu Kα = 0.15418 nm), *K* is 0.9 (Scherrer constant), *β* is FWHM and *θ* is diffraction angle (Bragg's).

The investigation of the composition of NACOs revealed intriguing findings through the utilization of ATR FT-IRS. Notably, ATR FT-IR peak observed at 995 cm^−1^ in NACO-5, 1022 cm^−1^ in NACO-10, and 1042 cm^−1^ in NACO-20 provides clear evidence of the presence of Ce–O in the samples (Fig. 1B). The shift towards higher wavenumbers with increasing Ce concentration indicates the influence of Ce on the vibrational behavior of the mixed oxides.^15^ This phenomenon may be ascribed to changes in the crystal structure and bonding environment resulting from the incorporation of Ce^4+^ ions into the lattice. Furthermore, another notable observation was the pronounced peak at 405 cm^−1^, signifying the formation of mixed oxides involving Ni, Al, and Ce.^16,17^ The appearance of this peak suggests the presence of intricate interactions and bonding arrangements among these elements, leading to the creation of distinct mixed metal oxides. The formation of these complex oxides may have important implications for the material's properties, such as adsorption, catalytic activity, electronic behavior, or structural stability.

BET analysis was implemented to assess the surface area and pore size of the produced NACO nanoparticles. Fig. 1C depicts the N_2_ adsorption–desorption isotherms for NACO-0 and NACO-20. It is evident from the graphs that both NACO-0 and NACO-20 revealed a type IV physisorption isotherm, signifying the existence of mesopores. This means that the nanoparticles have pores with widths between 2 and 50 nm. It is characterized by a sharp increase in adsorption at low relative pressures, followed by a plateau at higher relative pressures. This is due to the capillary condensation of nitrogen gas in the mesopores.^18^ The surface area and pore volume of NACO-20 were determined to be 138.89 m^2^ g^−1^ and 0.156 cm^3^ g^−1^, respectively (Table 2). Meanwhile, NACO-0 displayed a specific surface area of 130.77 m^2^ g^−1^, as shown in Table 2. It is reasonable to infer that the inclusion of Ce^4+^ in NACO could facilitate the penetration of N_2_ gas, resulting in a higher surface area. The addition of Ce^4+^ to NACO nanoparticles can increase the surface area by creating more defects and vacancies in the crystal lattice. These defects and vacancies can act as nucleation sites for N_2_ adsorption, which in turn increases the overall surface area of the material. Additionally, the Ce^4+^ ions can also help to stabilize the mesoporous structure of the nanoparticles, which further enhances N_2_ adsorption.^18^

**Table 2:** BET textural properties of NACOs

	Surface area (m^2^ g^−1^)	Pore volume (cm^3^ g^−1^)	Pore size (nm)
NACO-0	130.77	0.3	4.5
NACO-20	138.90	0.16	2.3

**Fig. 2:**
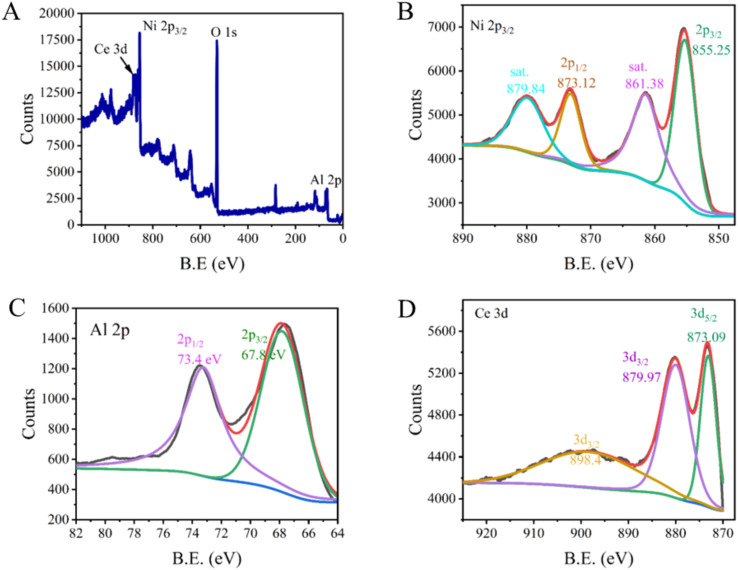
XPS spectrum of elements existing in NACO with their characteristic binding energies.

XPS was also used to ascertain the chemical states of elements present in NACO. The survey scan revealed the existence of Ni 2p, Ce 3d, Al 2p and O 1s, indicating the successful incorporation of these elements into the mixed oxide structure, while the negligible C 1s signal indicated the removal of carbonate during calcination of the precursor LDH (Fig. 2). The Ni 2p spectra exhibited two characteristic peaks at 855.25 eV (2p_3/2_) and 873.12 eV (2p_1/2_), with additional satellite peaks at 861.38 eV and 879.84 eV, which are indicative of the oxidation state Ni^2+^.^19^ Similarly, the Ce 3d spectra showed peaks at 873.09 eV (3d_5/2_) and 879.97 eV (3d_3/2_), in line with peak at 898.4 eV (3d_3/2_), suggesting the presence of Ce^4+^ in the mixed oxide.^20^ Furthermore, the Al 2p spectra displayed peaks at 67.8 eV (2p_3/2_) and 73.4 eV (2p_1/2_), consistent with the presence of Al^3+^ ions.^21^ These XPS results provided valuable insights into the oxidation states of the constituent elements within NACO mixed oxide, which is crucial for understanding its surface chemistry and potential as an efficient fluoride adsorbent.

**Fig. 3:**
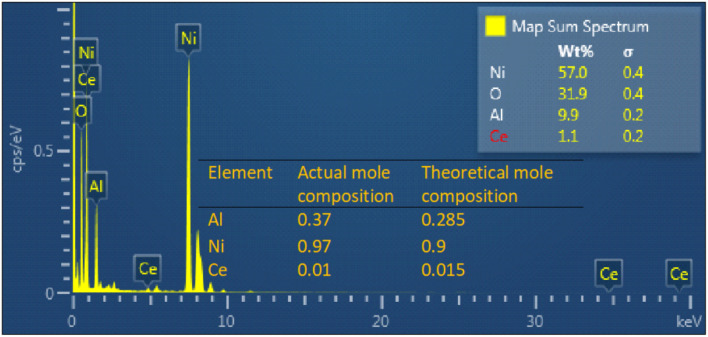
Percentage composition and mole ratio of elements in NACO-5 from EDS analysis spectra.

**Fig. 4:**
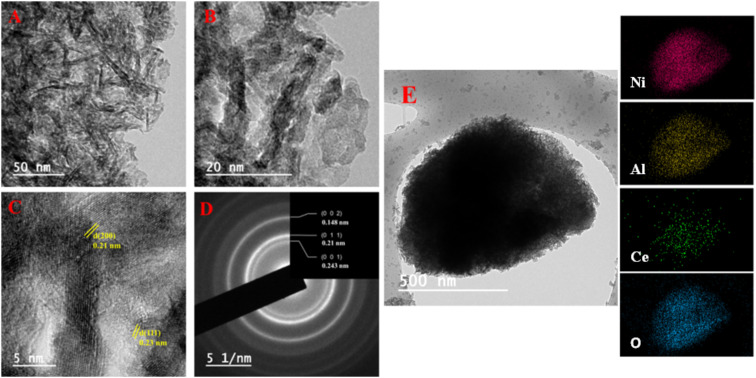
TEM images (A and B), HRTEM (C), SAED image (D) and TEM-EDS mapping (E) of NACO.

The TEM-EDS mapping analysis revealed a compelling demonstration of the uniform distribution of Ni, Al, Ce, and O elements within the NACO mixed metal oxide (Fig. 4E). This mapping technique allowed for a comprehensive visualization of the elemental composition across NACO, indicating a homogeneous dispersion of the aforementioned elements throughout the NACO structure. Notably, the absence of carbon in the synthesized NACO can be attributed to the effective removal of the layered double hydroxide (LDH) interlayer anion (CO_3_^2−^) during the calcination process. This observation is consistent with the XPS findings, thus reinforcing the reliability and coherence of the obtained results. Furthermore, the EDS provided the composition of Ce-doped Ni–Al MO. The mole ratios of the elements were calculated by dividing the weight percentage of each element by its atomic weight (Fig. 3). The actual mole composition of all the elements from the EDS analysis were found to be almost similar to the theoretical ones. Almost the intended amount of Ce was found in the yield of Ce-doped Ni–Al MO.

NACO mixed oxide was thoroughly characterized using HRTEM-SAED techniques (Fig. 4). The TEM analysis revealed the presence of aggregates exhibiting a sheet-like morphology, with 2.57 nm average size. The HRTEM measurements further confirmed the crystalline structure of the material by determining the values of *d*-spacing of 0.23 nm and 0.21 nm, which corresponded to (111) and (200) crystallographic planes, respectively. Notably, these values were found to be in excellent agreement with the results obtained from XRD analysis, corroborating the reliability of the characterization methods employed. Furthermore, the SAED analysis established the polycrystalline nature of NACO, indicating the presence of multiple crystal orientations within it. The *d*-spacing obtained from SAED are quite similar to that of HRTEM.

### 3.2 Influence of pH, dose, agitation and coexisting ions on fluoride adsorption

**Fig. 5:**
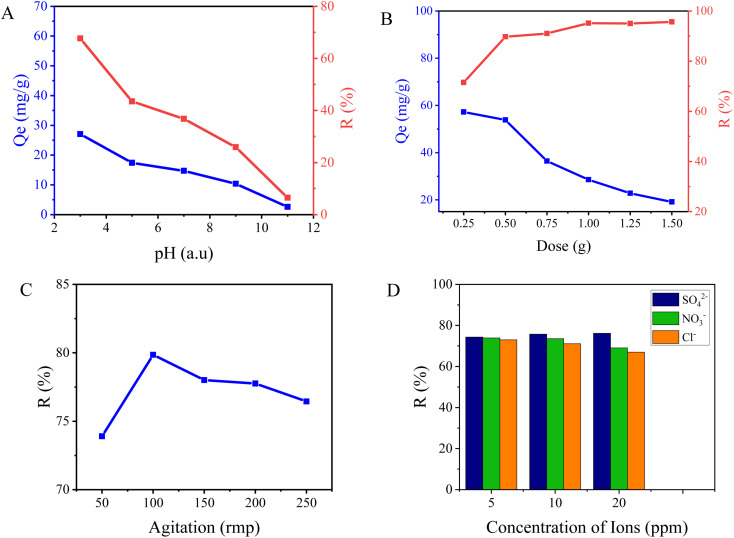
Graphs indicating influence of (A) pH, (B) dose, (C) agitation and (D) existence of other ions on fluoride adsorption performance of NACO.

In this study, the adsorption capacity and efficacy of fluoride were investigated by evaluating the performance of NACO at various pH levels ranging from 3 to 11 (Fig. 5A). The results revealed a clear trend in the fluoride adsorption capacity of NACO with changing pH, in which NACO-20 was identified as having better performance and was considered in studying all the adsorption parameters. At lower pH values of 3, NACO demonstrated a high adsorption capacity of 27.06 mg g^−1^, indicating its strong affinity for fluoride ions. However, as the pH increased, the adsorption capacity of NACO decreased significantly. The adsorption capacity dropped to 2.6 mg g^−1^ at pH 11, suggesting a reduced ability of NACO to effectively adsorb fluoride ions. Similarly, the removal efficiency exhibited a downward trend with increasing pH. At pH 3, NACO achieved a removal efficiency of 67.64%, indicating its excellent performance in removing fluoride from the solution. However, as the pH increased to 11, the removal efficiency decreased significantly to 6.49%, highlighting a diminished effectiveness of NACO in removing fluoride ions under alkaline conditions. This decrease in efficiency can be credited to the changing surface charge of NACO and the speciation of fluoride ions with varying pH values. At lower pH, the surface of NACO tends to be positively charged, favoring the electrostatic interaction and adsorption of fluoride ions. However, at higher pH, the surface charge becomes more negative, leading to repulsion between the negatively charged NACO surface and fluoride ions, resulting in decreased adsorption efficacy.

Another crucial factor is the impact of varying adsorbent amounts on fluoride adsorption using NACO. Six different concentrations of the adsorbent were employed, namely 0.25, 0.5, 0.75, 1.0, 1.25, and 1.5 g L^−1^ (Fig. 5B). In terms of adsorption capacity, the highest value was observed at the lowest adsorbent concentration, with a recorded capacity of 57.20 mg g^−1^. As the adsorbent amount increased gradually, the adsorption capacity experienced a significant decline reaching a capacity of 19.13 mg g^−1^ at 1.5 g L^−1^. The experimental reduction in adsorption capacity might be ascribed to the limited accessibility of active sites on NACO surface as its amount increased. However, the highest removal efficiency, about 95%, was achieved at an adsorbent concentration of 1.0 g L^−1^. After reaching this highest peak the removal efficiency remained constant.

The consequence of agitation speed on the adsorption of fluoride ions using NACO mixed metal oxide was also investigated. The experiment aimed to evaluate how varying agitation speeds influenced the adsorption capacity and efficiency of fluoride ions onto the synthesized mixed metal oxide. At an agitation speed of 50 rpm, the adsorption capacity was measured as 29.56 mg g^−1^, while at 100 rpm, it increased to 31.94 mg g^−1^ (Fig. 5C). Further increase in agitation speed to 150 rpm resulted in a slight decrease in adsorption capacity to 31.20 mg g^−1^. At 200 rpm and 250 rpm, the adsorption capacities were measured as 31.10 mg g^−1^ and 30.58 mg g^−1^, respectively. Similarly, the removal efficiency of fluoride ions demonstrated a similar pattern. At 50 rpm, the removal efficiency was 73.90%, which increased to 79.85% at 100 rpm. At 150 rpm, the removal efficiency slightly decreased to 78.01%. At 200 rpm and 250 rpm, the removal efficiencies were measured as 77.76% and 76.45%, respectively. These findings can be ascribed to the accessibility of active sites on the surface of NACO, mass transfer limitations, and the level of agitation-induced mixing. Lower agitation speeds may result in slower diffusion of fluoride ions to the active sites, leading to relatively lower adsorption efficiencies. Conversely, higher agitation speeds enhance the diffusion process, thereby improving the adsorption efficiencies. It is worth noting that excessive agitation can potentially cause desorption of adsorbed fluoride ions from the surface of NACO which may be due to attrition of the adsorbent and mechanical impacts at high agitation speed. This detachment can subsequently reduce the overall adsorption efficiency, as observed at the highest agitation speed of 250 rpm.^22^

The effect of coexisting ions which are commonly found in drinking water, namely SO_4_^2−^, NO_3_^−^, and Cl^−^, on the adsorption of F^−^ by Ce-doped Ni–Al MO was also studied in the present work. The removal efficiencies of F^−^ were investigated at different concentrations of these coexisting ions, namely 5, 10, and 20 ppm, in the presence of 20 mg per L F^−^ (Fig. 5D). The results showed that the presence of coexisting ions influenced the removal efficiency of F^−^. In the presence of SO_4_^2−^ ions at concentrations of 5, 10, and 20 ppm, removal efficiencies of 74.3%, 75.72%, and 76.18% were obtained, respectively. Similarly, in the presence of NO_3_^−^ ions at concentrations of 5, 10, and 20 ppm, removal efficiencies of 73.90%, 73.50%, and 69.03% were obtained, respectively. Finally, in the presence of Cl^−^ ions at concentrations of 5, 10, and 20 ppm, removal efficiencies of 73%, 71.08%, and 66.97% were obtained, respectively. These results indicated that the coexisting ions can slightly affect the removal efficiency of fluoride, with varying degrees of influence depending on the specific ion. Ce^4+^ can interact differently with various anions depending on the solution's pH. The pH influences the speciation of the anions and the availability of Ce^4+^ for interaction.^23^

### 3.3 Investigation of fluoride adsorption kinetics

**Fig. 6:**
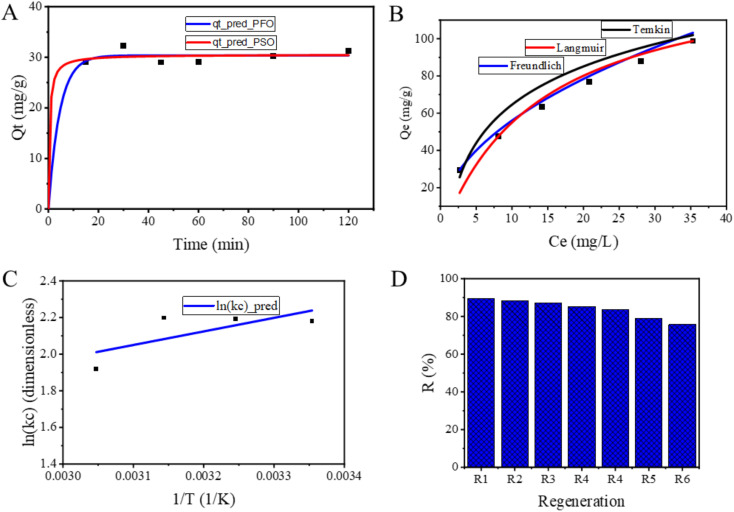
Graphs of (A) kinetics, (B) isotherms and (C) van't Hoff fitted to fluoride adsorption data and (D) reuse performance of NACO for fluoride adsorption.

The adsorption kinetics of fluoride onto NACO was examined by varying the contact time from 15 to 120 min at initial F^−^ concentration of 20 mg L^−1^, pH of 3 and temperature of 25 °C. To analyze the kinetics of adsorption process, nonlinear pseudo-first order (PFO) and pseudo-second order (PSO), were employed (Tables 3, 4 and Fig. 6A).

**Table 3:** Comparisons of *q*_t_pred_ from kinetic models with *q*_t_exp_

Time (min)	*q*_t_exp_ (mg g^−1^)	*q*_t_pred_ (mg g^−1^), PFO	*q*_t_pred_ (mg g^−1^), PSO
0	0	0	0
15	29.13	29.27	29.63472
30	32.33	30.33	30.07939
45	29.00	30.36	30.23059
60	29.13	30.37	30.30676
90	30.20	30.37	30.38332
120	31.26	30.37	30.42174

**Table 4:** Outputs of kinetics and isotherm models fitted to results from adsorption of fluoride onto NACO

Types of models	Parameters and values					
PFO	*q*_e_ (mg g^−1^)	30.37	*K*_1_ (1/min)	0.22	*R* ^2^	0.99
PSO	*q*_e_ (mg g^−1^)	30.54	*K*_2_ (g mg^−1^ min^−1^)	0.07	*R* ^2^	0.989
Freundlich	*K*_f_ ((mg g^−1^) (L mg^−1^)^1/*n*^)	17.75	*N* (dimension-less)	2.08	*R* ^2^	0.999
Langmuir	*q*_max_ (mg g^−1^)	132	*K*_L_ (L mg^−1^)	0.07	*R* ^2^	0.97
Temkin	*K*_T_ (J mg^−1^)	0.92	*B*_T_ (L mg^−1^)	92.9	*R* ^2^	0.962

The Table 3 showed that the *q*_t_pred_ data from both methods is generally close to the *q*_t_exp_ data. However, there are some differences between the two methods. For example, the *q*_t_pred_ data from the PFO method is slightly lower than the *q*_t_exp_ data at the early time points (15 and 30 min), while the *q*_t_pred_ data from the PSO method is slightly higher than the *q*_t_exp_ data at the later time points (90 and 120 min). Even though the results suggest that the *q*_t_pred_ data from PFO is slightly closer to the *q*_t_exp_ data than the *q*_t_pred_ data from PSO, both methods are able to predict the *q*_t_exp_ data reasonably well.

For PFO model, *q*_e_ was determined to be 30.37 mg g^−1^. Furthermore, the rate constant (*K*_1_) associated with the PFO model was found to be 0.22, indicating the speed at which the adsorption process occurs (Table 4). The goodness of fit for this model was assessed using *R*^2^, yielding an impressive value of 0.99. This high *R*^2^ value suggests that PFO model effectively describes the adsorption kinetics of F^−^ onto NACO.

Similarly, *q*_e_ obtained from the PSO model was determined to be 30.54 mg g^−1^, indicating a slight increase compared to the PFO model (Table 4). The rate constant (*K*_2_) associated with the PSO model was found to be 0.07, suggesting a relatively slower adsorption process compared to the PFO model. The PSO model's goodness of fit was evaluated using the *R*^2^, which yielded a value of 0.989, indicating a strong correlation between the model and the experimental data (Fig. 6A). In conclusion, the results demonstrated that both models were able to describe the adsorption process effectively, as evidenced by high *R*^2^ values. This means that the process is influenced by both mass transfer and chemical reaction that takes place between the adsorbent and adsorbate.^24^

### 3.4 Isotherms of fluoride adsorption

The adsorption behavior of fluoride onto NACO was investigated by examining the adsorption isotherms at various initial F^−^ concentrations ranging from 10 to 60 ppm. The obtained data was analyzed using different nonlinear isotherm models, namely Freundlich, Langmuir and Temkin (Tables 4, 5 and Fig. 6B).

**Table 5:** Comparisons of *q*_eq_pred_ from isotherm models with *q*_t_exp_

*C*_eq_ (mg L^−1^)	*q*_eq_exp_ (mg g^−1^)	*q*_eq_pred_ (mg g^−1^), Freundlich	*q*_eq_pred_ (mg g^−1^), Langmuir	*q*_eq_pred_ (mg g^−1^), Temkin
2.68	29.28	28.52	21.46	23.97
8.07	47.72	48.45	48.69	53.38
14.16	63.34	63.51	66.85	68.39
20.82	76.71	76.44	79.38	78.67
28.02	87.93	88.17	88.43	86.59
35.30	98.78	98.53	94.89	92.76

As shown in Table 5, the error between the *q*_eq_exp_ values and the *q*_eq_pred_ values is greater for the Temkin model than for the other models and is least for the Freundlich model. This suggested that the Freundlich model was the best-fitting model, as it has the lowest overall error between the *q*_eq_exp_ values and the *q*_eq_pred_ values.

For the Freundlich isotherm model, the obtained parameters were *K*_f_ = 17.75 and *N* = 2.08, with a high coefficient of determination (*R*^2^) of 0.999 (Table 4). The Freundlich model suggests that F^−^ adsorption onto NACO is favorable and follows a heterogeneous process.^25^

The Langmuir model yielded a *q*_max_ of 132 mg g^−1^, and a Langmuir constant (*K*_L_) of 0.07, with *R*^2^ value of 0.97 (Table 4). The Langmuir model assumes adsorption of monolayer type onto a homogeneous surface, indicating that the adsorption of fluoride onto NACO follows a Langmuir-type mechanism.^26^

The Temkin isotherm model resulted in a Temkin constant (*K*_T_) of 0.92 and a Temkin heat of adsorption (*B*_T_) of 92.9, along with *R*^2^ value of 0.962 (Table 4). The Temkin model implies a weaker adsorbent–adsorbate interaction, suggesting that the adsorption of fluoride onto NACO involves a combination of physical and chemical interactions.^27^

In summary, these isotherm models provide valuable insights into the adsorption behavior of fluoride onto the synthesized NACO material. The high *R*^2^ values obtained indicate a satisfactory match of the experimental data to Freundlich model, implying that the adsorption process is heterogeneous. These results suggest that NACO has a high affinity for fluoride and could be promising adsorbent for water treatment applications targeting fluoride removal.

### 3.5 Thermodynamics of adsorption of fluoride

The consequence of temperature on the adsorption of fluoride onto NACO was investigated in this study. The thermodynamic parameters, including the Gibbs free energy change (Δ*G*), dimensionless equilibrium constant (*K*_c_), enthalpy change (Δ*H*) and entropy change (Δ*S*), were estimated using van't Hoff fitting based on the equilibrium constants obtained from the adsorption experiments (Fig. 6C). The data at different temperatures are summarized in Table 6. The Δ*G* with negative values indicates that F^−^ adsorption onto NACO is spontaneous at the studied temperatures. The magnitude of Δ*G* suggests the thermodynamic favorability of the adsorption and that NACO has a high affinity for fluoride ions. The negative Δ*H* values indicate the adsorption process is exothermic, implying that the adsorption is driven by heat release. The positive Δ*S* values suggest an increase in the randomness of the system during the adsorption course, possibly due to the rearrangement of F^−^ or changes in the adsorbent's surface as it interacts with F^−^.^28^

**Table 6:** Thermodynamic outputs of fluoride adsorption onto NACO at various temperatures

*K*_c_ (dimensionless)	*T* (°C)	Δ*G* (kJ mol^−1^)	Δ*H* (kJ mol^−1^)	Δ*S* (J mol^−1^)
8.59	25	−5.33	−5.46	0.13
8.97	35	−5.62		
9.01	45	−5.81		
6.81	55	−5.23		

The obtained data provide insights into the thermodynamic parameters governing the adsorption process and shed light on the feasibility and spontaneity of fluoride adsorption onto NACO at different temperatures. This information is valuable for understanding the underlying mechanisms of fluoride adsorption and for optimizing adsorption processes in water treatment applications.

### 3.6 Investigation of regeneration and reusability

A series of regeneration cycles were conducted to evaluate the reusability performance of NACO nanomaterial for adsorbing fluoride from aqueous solutions. The experimental setup involved using 0.5 g L^−1^ of NACO nanomaterial as adsorbent and an initial fluoride amount of 20 mg L^−1^. The removing efficiencies for the first six regeneration cycles were determined to be 89.39%, 88.33%, 87.023%, 85.23%, 83.39%, 78.9%, and 75.71%, respectively (Fig. 6D).

These results indicate that NACO nanomaterial possesses excellent regeneration capabilities, allowing for repeated usage without significant loss in adsorption efficiency. The high removal efficiencies obtained in the subsequent cycles suggest that the nanomaterial maintains its adsorption capacity even after multiple regeneration cycles. This finding is of great significance for practical applications, as it highlights the potential of NACO nanomaterial as a cost-effective and sustainable adsorbent for F^−^ removing from aqueous solutions.

### 3.7 Adsorption mechanism of F^−^ onto NACOs

The adsorption of fluoride ions (F^−^) onto Ce^4+^-doped Ni–Al MOs is a complex process that involves multiple factors, including the electrostatic interaction between the positively charged ions (Ni^2+^, Al^3+^, and Ce^4+^) and the negatively charged F^−^ ions, the formation of complexes between positive ions and F^−^, and the memory-effect/reconstruction of MOs to LDHs.^29–31^

The electrostatic interaction between F^−^ ions and Ce^4+^-doped Ni–Al MOs is indeed the primary driving force for adsorption. The positive charge of the Ce^4+^, Ni^2+^, and Al^3+^ ions in MOs attract the negatively charged F^−^ ions. The strength of this interaction depends on the pH of the solution. At low pH, the surface of the MOs is protonated, which increases the positive charge of the surface and strengthens the interaction. As the pH increases, the surface of MOs becomes deprotonated, which reduces their positive charge and weakens the electrostatic interaction with F^−^.^30,31^

The formation of complexes between NACOs and F^−^ is another important mechanism in the adsorption of F^−^ onto NACOs. The complexes are formed when a ligand, such as F^−^, shares an electron pair with a metal ion, such as Ce^4+^, and Al^3+^. The formation of complexes is favored at low pH, where the positive ions are more likely to be hydrated. Hydrated ions have a larger radius than anhydrous ions. This means that the hydrated cations are more likely to be able to form complexes with F^−^ ions. These complexes can help to hold the F^−^ ions onto the surface of the material.^29,31^

The complex formation can be explained in terms of Lewis acid–base interaction between positive ions of NACOs and F^−^. This is because for instance Ce^4+^ is a Lewis acid, which means it has an empty orbital that can accept electron pairs, whereas F^−^ is a Lewis base, which means it has a lone pair of electrons that it can donate.

The reconstruction of the Ce-doped Ni–Al mixed oxides into a hydrotalcite-like structure during adsorption can also contribute to the high adsorption capacity. Fluoride ions are strongly adsorbed and intercalated into the interlayers of the reconstructed hydrotalcite-like sheets, enhancing the overall adsorption capacity.^32^

**Fig. 7:**
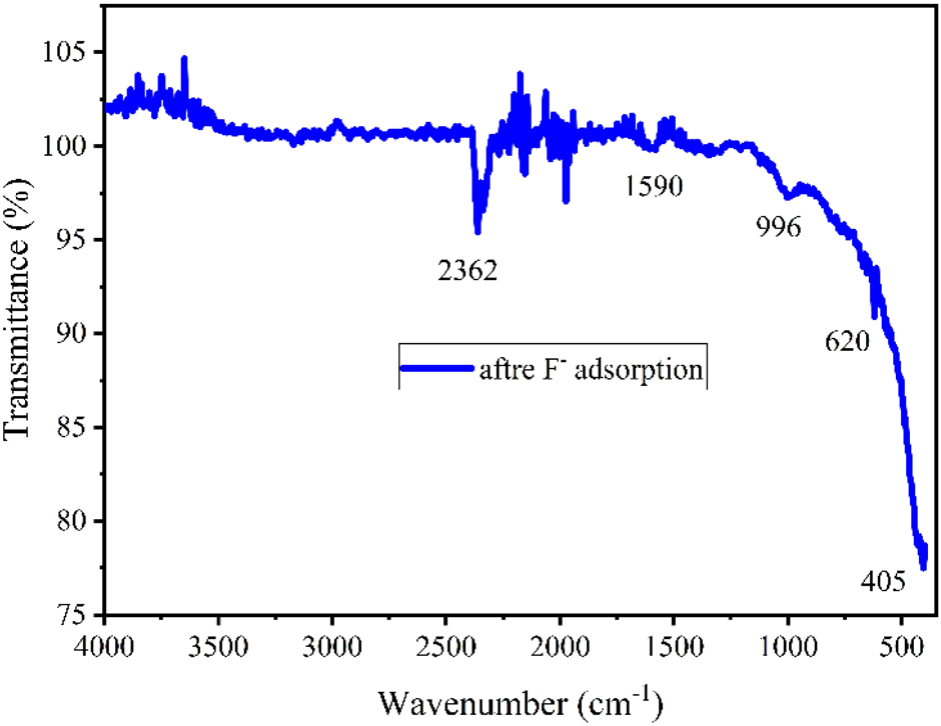
ATR-IR of exhausted Ce-doped Ni–Al mixed oxide.

ATR FT-IR study provided evidence to support the proposed mechanisms for F^−^ adsorption by NACOs as the emergence of intensified peaks between 2400 to 1500 cm^−1^, and a new peak at around 620 cm^−1^ in the exhausted material (Fig. 7). The presence of intensified peaks in the spectrum of the exhausted material compared to the pristine material indicated changes in the chemical environment of the material. These changes could be due to the interaction between the F^−^ ions and the surface of the NACOs. Additionally, the presence of new peaks suggested the formation of new chemical species as a result of the adsorption process.^30,31^

## 4. Conclusions

NACO with sheet-like morphology and nodular appearance was successfully synthesized. The XRD analysis confirmed the formation of mixed oxides with NaCl-type crystal structure. TEM-EDS mapping and XPS analysis verified the presence of Ni, Al, Ce, and O elements in different oxidation states. The NACO exhibited type IV physisorption isotherm behavior according to BET analysis. Both PFO and PSO kinetic models fitted well with the experimental data. The Freundlich isotherm model showed excellent fit (high *R*^2^ values). Langmuir model revealed a *q*_max_ of 132 mg g^−1^ for fluoride. Thermodynamics indicated the feasibility and spontaneity of fluoride adsorption onto NACO. Remarkably, NACO demonstrated a high fluoride removal efficiency of 75.71% even after multiple regeneration cycles, highlighting its promising potential for fluoride adsorption applications.

Overall, these findings establish Ce^4+^-doped Ni–Al mixed oxides as a promising and efficient adsorbent material for fluoride removal, opening up possibilities for its application in water treatment and environmental remediation strategies. Further research could focus on optimizing the synthesis parameters and exploring other dopants to further enhance the material's performance.

## Author contributions

Ararso Nagari Wagassa: conceptualization, data curation, formal analysis, investigation, methodology, software, writing – original draft. Amit Bansiwal: supervision, funding acquisition, project administration, resources, validation, writing – review & editing. Tofik Ahmed Shifa: supervision, conceptualization, validation, writing– review & editing. Enyew Amare Zerffa: supervision, conceptualization, validation, writing – review & editing descriptions.

## Conflicts of interest

The authors of this paper assert that they have no known competing issues that could have potentially impacted the research findings presented in this study.

## Supplementary Material
